# Renal Hydatid Cyst: A Report of Two Cases

**DOI:** 10.7759/cureus.106102

**Published:** 2026-03-30

**Authors:** Ali Akjay, Najwa Jmil, Akdad Mourad, Jihad El Anzaoui

**Affiliations:** 1 Urology, Faculty of Medicine, Université Sidi Mohamed Ben Abdellah, Fez, MAR; 2 Urology, Military Hospital Moulay Ismail, Meknes, MAR; 3 Ethnopharmacology and Pharmacognosy, Faculty of Sciences and Techniques, Moulay Ismail University of Meknes, Errachidia, MAR; 4 Urology, Faculty of Medicine, Université Sidi Mohamed Ben Abdellah, Meknes, MAR

**Keywords:** echinococcus granulosus, hydatidosis, hyperechoic cysts, pericystomy, recidivism, renal hedratic cyst, tapeworm

## Abstract

Hydatidosis is an endemic, widely distributed anthropozoonosis that involves the liver, lungs, and other organs. We report two cases of large renal hydatid cysts. Diagnosis was suspected based on radiological examinations. The patients were given albendazole tablets 400 mg twice per day (one month before and one month after surgery), with monitoring of blood count and liver enzymes. During follow-up assessment for renal hydatid cysts, the possibility of recurrence must be kept in mind. These cysts can completely destroy the kidney.

## Introduction

Hydatid disease (hydatidosis) is a cosmopolitan parasitic anthropozoonosis caused by the larval stage of the tapeworm Echinococcus granulosus. It remains a significant public health issue in many sheep-raising regions, including the Mediterranean basin, North Africa (notably Morocco, Tunisia, and Mauritania), Australia, and New Zealand [1-3]. Humans act as an accidental intermediate host, becoming contaminated through the ingestion of food or water soiled with cestode eggs from dog feces [4-6]. While the liver and lungs are the most common sites for hydatid cysts, the disease can affect virtually any part of the body.

Renal hydatidosis is a relatively rare condition, representing only 2-4% of all visceral localizations, which places it third in frequency after hepatic and pulmonary involvement [2-4]. The kidney is the most frequently affected organ in the urogenital tract [1]. The cyst is typically solitary, unilateral, and develops slowly in the retroperitoneal space, often remaining clinically silent for a long time. Consequently, the diagnosis is often delayed, and the cyst can reach a considerable size before becoming symptomatic. When symptoms do occur, they are often nonspecific, with pain being the most common presenting complaint [4,7,8]. Hydaturia (the passage of daughter vesicles in the urine) is the only pathognomonic sign, but it is rare [1].

Diagnosis relies primarily on imaging. Ultrasound, with its high reliability, is the cornerstone of diagnosis, and findings are typically classified according to the Gharbi classification [2,4]. Computed tomography (CT) is invaluable for confirming the diagnosis, especially in complex cases. It helps differentiate a hydatid cyst from a renal tumor by demonstrating the absence of contrast enhancement, precisely assessing the cyst's relationship with surrounding structures, evaluating the remaining healthy parenchyma, and detecting potential communications with the excretory tract [9,10]. Serological tests and hypereosinophilia can be supportive but are neither sensitive nor specific enough to be diagnostic on their own [2,11].

The management of renal hydatid cysts presents a therapeutic challenge. While surgery remains the treatment of choice, the approach must balance the goal of cyst removal with the preservation of renal function. Options range from conservative procedures like pericystectomy or resection of the protruding dome to radical nephrectomy, which is reserved for cases where the kidney has been destroyed by a large cyst [7,12,13]. Medical treatment with albendazole is generally considered an adjuvant to surgery to reduce the risk of recurrence, especially in complex cases [14,15].

We report here two cases of large renal hydatid cysts in patients from Morocco. These cases are particularly noteworthy due to the extreme size of the cysts (14 cm and 17 cm), which had destroyed the functional parenchyma, necessitating radical nephrectomy. Through these cases, we aim to highlight the clinical presentation, diagnostic imaging features, and surgical management of this rare entity, as well as to underscore the importance of considering hydatid disease in the differential diagnosis of complex renal cysts in endemic regions.

## Case presentation

Case 1

A 36-year-old male patient presented to the clinic with a six-month history of right lower back pain. He had no history of diabetes, hypertension, or immunosuppression, and resided in a rural area where sheep farming was the primary economic activity, and stray dogs were common. The patient had undergone surgery for a renal hydatid cyst (pericystectomy) 10 years ago. Renal function was normal, and hydatid serology was negative.

**Figure 1:**
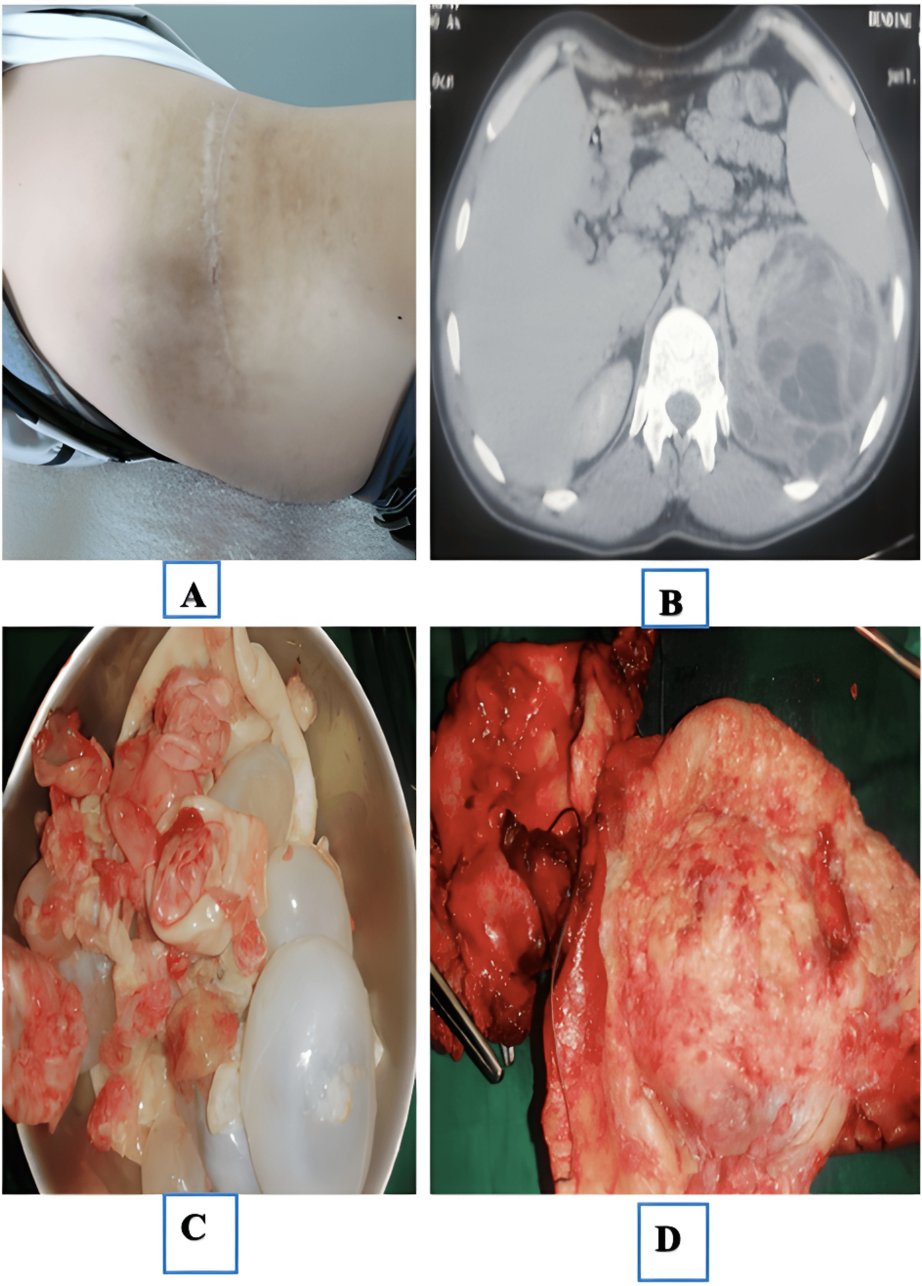
(A) Left flank surgical scar from previous hydatid surgery; (B) CT scan showing multilocular cystic lesion in left kidney; (C) Proligerous membrane of hydatid cyst; (D) Surgical specimen following radical nephrectomy

Clinical examination revealed a left flank surgical scar (Figure 1A) and a firm mass occupying almost the entire left side of the abdomen. Ultrasound identified multiple hyperechoic cysts of various sizes located in the left kidney. CT finally revealed a large, heterogeneous, multilocular cyst measuring 14 × 6 cm, which did not enhance with contrast (Figure 1B). The cyst wall was thin with no evidence of nodular thickening. Multiple internal septa and internal daughter cysts were present, without perceptible contrast enhancement or calcifications. These findings were most consistent with a Bosniak category III cyst.

An incision was made over the 11th rib, and the retroperitoneal space was accessed. A radical nephrectomy was performed in the plane of the psoas muscle after control of the renal pedicle was achieved. During surgery, the operating field was protected with scolicidal agents. Preoperative albendazole (400 mg twice daily) was administered for two weeks to reduce cyst viability and intracystic pressure, followed by a three-month postoperative course to minimize the risk of secondary hydatidosis. To ensure parasitic safety, the operative field was first protected with gauze pads soaked in 10% povidone-iodine to establish a scolicidal barrier. Following a careful incision of the renal capsule over the cyst wall, controlled aspiration was performed to decompress the lesion. Hypertonic saline (20%) was then injected into the cavity and maintained for 10 minutes to ensure scolicidal efficacy. Finally, the cyst was opened, allowing for the evacuation of the laminated membranes and daughter cysts, while strictly avoiding any spillage into the retroperitoneal space (Figure 1C, 1D).

The procedure was performed without any perioperative complications; notably, there was no occurrence of anaphylactic shock. The patient did not require a blood transfusion. His postoperative course was unremarkable; he recovered well and was successfully discharged on postoperative day 4. To monitor for potential complications or relapse, follow-up renal ultrasonography was performed at one and three months. These scans confirmed no evidence of recurrence, indicating a successful surgical outcome.

Histopathological examination of the surgical specimen confirmed the diagnosis of cystic echinococcosis, revealing the pathognomonic acellular laminated membrane in both cases. Hydatid serology was negative, and follow-up imaging showed no evidence of recurrence.

Case 2

A 65-year-old male patient, who lived in a rural area and had no significant medical history, presented with right-sided lower back pain of four months' duration.

Preoperative imaging revealed a large, multilocular cyst measuring 15 × 8 cm at the lower pole of the left kidney (Figure 1A). The cyst wall was thin with no evidence of nodular thickening. Multiple internal septa and internal daughter cysts were present, without perceptible contrast enhancement or calcifications. These findings were most consistent with a Bosniak category III cyst. Lymphocyte counts were normal, and hydatid serology was negative. Dimercaptosuccinic acid (DMSA) renal scintigraphy showed a non-functioning right kidney.

**Figure 2:**
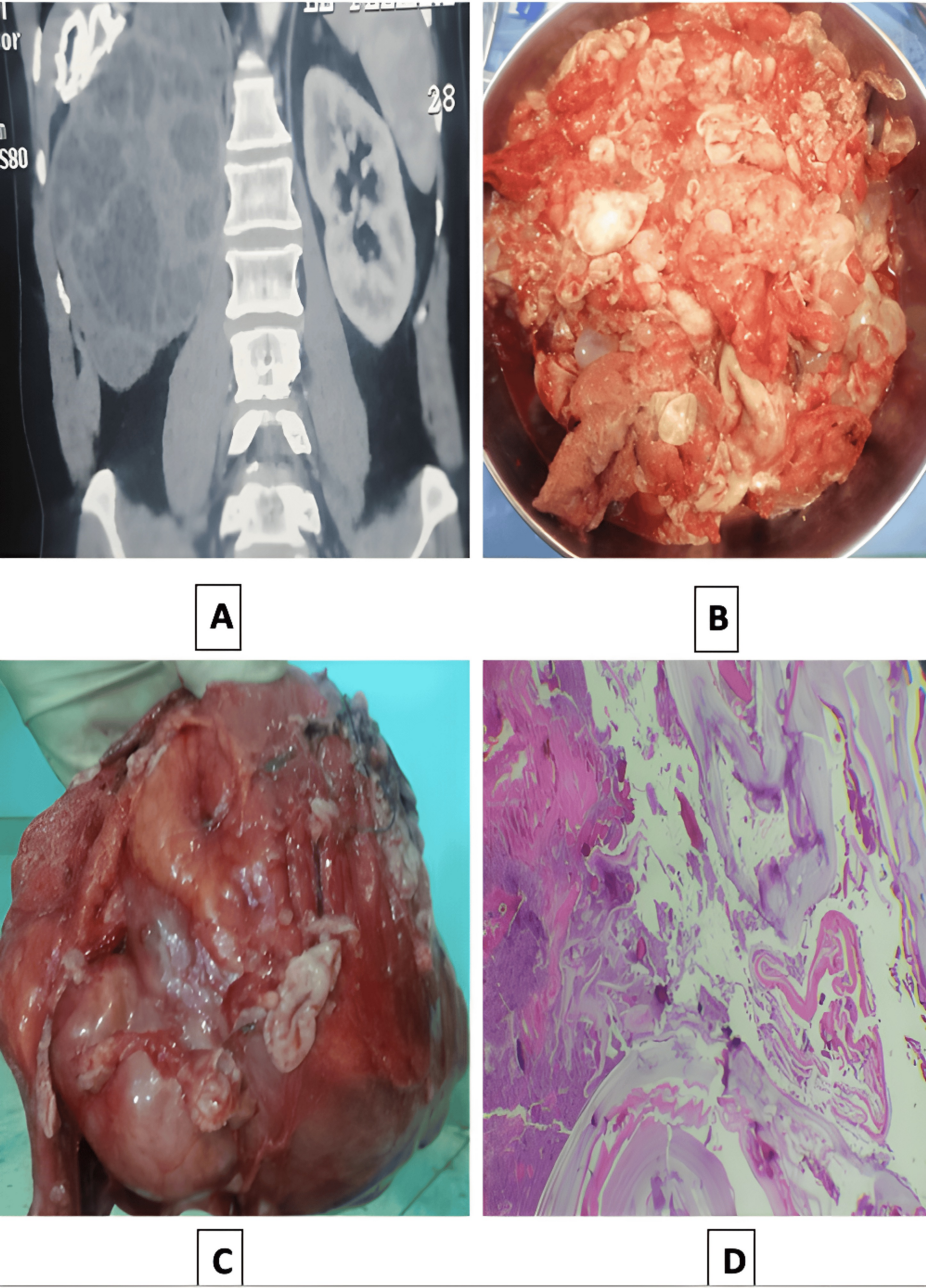
(A) Abdominopelvic CT scan showing large, heterogeneous, multilocular cystic mass, measuring 11 × 7 cm, occupying the region of the right kidney, not enhancing with contrast; (B) Vesicles of a multivesicular hydatid cyst; (C) Intact extended nephrectomy surgical specimen measuring 13x11x8 cm; (D) Protoscolex under fluorescence microscopy

The patient underwent total nephrectomy via open surgery (same as the first patient) (Figure 2B, 2C). The postoperative course was uneventful, and he was discharged on day 5. Histopathological analysis confirmed cystic echinococcosis, despite negative hydatid serology (2D). Follow-up imaging demonstrated no signs of recurrence, confirming a successful surgical and clinical outcome.

## Discussion

Hydatidosis is a cosmopolitan parasitic disease, encountered especially in countries where traditional sheep farming remains prevalent. It is found particularly in Australia, New Zealand, North Africa, and around the Mediterranean basin, including Morocco, where it constitutes a real public health problem [1]. In Morocco, the annual incidence of hydatidosis is 6.4 per 100,000 inhabitants [16]; in Tunisia, 15 per 100,000 [17]; and in Mauritania, 1.2 per 100,000 [3]. Renal hydatid cyst accounts for 2-3% of visceral hydatid cysts, and the kidney is the third most common location, after hepatic and pulmonary locations [2-4]. Humans represent a biological dead-end in the evolutionary cycle of *E. granulosus*. Infection occurs through the ingestion of food contaminated with cestode eggs from dog feces or through direct contact with an infected dog [5,6]. According to some studies, this pathology predominantly affects young adults, with a mean age ranging from 36 to 49.5 years [4,6]. In both of our cases, the mean age at diagnosis was 55.5 years.

Renal hydatid cyst is typically solitary and unilateral, with a predilection for the left kidney. Due to its retroperitoneal development, the renal hydatid cyst is characterized by its clinical latency. Pain is the main presenting symptom, occurring in 62.8-90% of cases [4,7]. Other signs, such as fever, hypertension, and urinary symptoms, remain rare. Hydatiduria is the only pathognomonic sign, reflecting the rupture of a hydatid cyst into the excretory tract [1].

The kidney is the most common site of urogenital tract hydatid cysts. A renal hydatid cyst is often primary, almost always solitary, typically unilateral, and located in the renal cortex and the subcapsular space. Radiological assessment remains the essential element in establishing the diagnosis. According to Hetet et al., a plain abdominal radiograph of the urinary tract may reveal calcifications in the renal area in 15-60% of cases [8]. These calcifications are suggestive but not specific, as they are seen in 2% of serous cysts and in 10-15% of malignant tumors. According to some authors, the presence of calcifications does not necessarily indicate the death of the cyst [8]. Ultrasound is reliable in approximately 80% of cases, even when the cyst has ruptured into the excretory tract [18]. The different sonographic appearances are classified according to the Gharbi classification system (Table 1). In our observation, the ultrasound appearance corresponded to Gharbi stage I [19].

**Table 1:** Gharbi classification Reference: Horchani et al., 1983 [19]

Type	Description
Type I	Pure fluid mass with own wall
Type II	Fluid collection with membrane detachment
Type III	Cloisonne, multivesicular collection
Type IV	Heterogeneous mass with a pseudotumor appearance
Type V	Calcified wall

CT is generally requested when diagnostic doubt arises on ultrasound, particularly for Gharbi types I and IV, or in the presence of complications. It allows for the detection of calcifications and helps determine the nature of the renal mass by distinguishing between a pseudotumoral hydatid cyst and a renal tumor, based on the absence of cyst wall enhancement after contrast injection. It also specifies the exact location of the cyst and its relationship to surrounding structures, and allows for the detection of communication between the cyst and the excretory tract, which is demonstrated by opacification of the cystic cavity following contrast injection during the excretory phase of acquisition. CT also enables an assessment of the remaining healthy parenchyma for possible conservative surgery, as well as the detection of other hydatid locations [9,10].

Hypereosinophilia is neither a specific nor a constant finding, and is present in only 20-50% of cases, depending on the series [2,11]. Regarding the therapeutic approach, the general recommendations are less applicable to renal hydatid cysts than to hepatic hydatid cysts. Surgery is widely considered the treatment of choice for renal hydatid cysts. For many surgical teams, pericystectomy (also referred to as dome resection) is the preferred surgical method, as it is simple, quick to perform, and associated with fewer postoperative complications [7,12,13]. This procedure involves resecting the exteriorized, superficial, and avascular portion of the pericyst. It is typically performed when the walls of the residual cavity are pliable and non-fibrotic.

Resection of the protruding dome yields excellent results and allows for good re-expansion of the renal parenchyma [8,12,13]. Total pericystectomy is often difficult to perform due to the risk of bleeding or communication with the excretory tract. Partial nephrectomy is recommended by some authors for large cysts. However, for most surgical teams, this approach seems unjustified. Total nephrectomy, for its part, is reserved only for cases where the kidney has been destroyed by a large renal hydatid cyst or in the presence of major suppuration. 

Laparoscopic surgery has been successfully used in recent years for the treatment of renal hydatid cysts. Both transperitoneal and retroperitoneal approaches have been attempted. However, the transperitoneal route carries a risk of disseminating cyst contents into the peritoneal cavity [14]. Despite its proven efficacy, percutaneous treatment of hydatid cysts does not meet with unanimous approval. One of the main reasons for this reluctance is the risk of an anaphylactic reaction that it entails [15]. 

According to most authors, medical treatment alone is insufficient, and its effectiveness remains controversial. It is indicated in patients who are inoperable or who have multiple cysts [15]. Therapeutic abstention may be indicated in elderly patients with a small, non-progressive, asymptomatic cyst and a low level of specific immunoglobulins. The effectiveness of medical treatment alone remains controversial and is indicated in cases of multiple hydatid locations, incomplete surgical resection, or when associated with diffuse peritoneal involvement [7,15].

The differential diagnosis of a renal hydatid cyst primarily includes complex renal cysts (Bosniak III/IV) and cystic renal cell carcinoma, which frequently share overlapping features such as internal septations and wall thickening. However, pathognomonic radiological signs, such as daughter cysts or the "double-rim" sign, typically differentiate hydatid disease from these malignant or complex cystic lesions. Inflammatory processes, specifically renal abscess and xanthogranulomatous pyelonephritis (XGP), should also be entertained, especially when associated with staghorn calculi or systemic signs of infection. Accurate preoperative identification of these hallmarks remains vital to preclude contraindicated percutaneous biopsies and the subsequent risk of anaphylactic shock [20].

## Conclusions

Renal hydatid cyst remains a rare but important differential diagnosis of complex renal cysts in endemic regions. Early recognition through imaging may allow renal-preserving surgery, while radical nephrectomy may be necessary in cases with extensive parenchymal destruction.
